# Diversity of fecal parasitomes of wild carnivores inhabiting Korea, including zoonotic parasites and parasites of their prey animals, as revealed by 18S rRNA gene sequencing

**DOI:** 10.1016/j.ijppaw.2023.05.005

**Published:** 2023-06-03

**Authors:** Cheolwoon Woo, Mohammad Imtiaj Uddin Bhuiyan, Kyung Yeon Eo, Woo-Shin Lee, Junpei Kimura, Naomichi Yamamoto

**Affiliations:** a Department of Environmental Health Sciences, Graduate School of Public Health, Seoul National University, Seoul, 08826, Republic of Korea; b Department of Animal Health and Welfare, College of Healthcare and Biotechnology, Semyung University, Jecheon, 27136, Republic of Korea; c Department of Forest Sciences, College of Agriculture and Life Science, Seoul National University, Seoul, 08826, Republic of Korea; d College of Veterinary Medicine, Seoul National University, Seoul, 08826, Republic of Korea; e Institute of Health and Environment, Graduate School of Public Health, Seoul National University, Seoul, 08826, Republic of Korea

**Keywords:** Amoeba, DNA barcoding, Nematode, Platyhelminthes, *Enterocytozoon bieneusi*, *Capillaria hepatica*

## Abstract

Consisting of diverse groups of organisms, parasites are among the least studied pathogens despite their enormous impacts on humans, livestock, and wildlife. In particular, little is known about their host specificity and diversity in wildlife. Here, using multiple primer pairs and sequencing 18S rRNA genes of diverse groups of parasites, we aimed to investigate fecal parasitomes of carnivorous wildlife in Korea, namely, the raccoon dog (*Nyctereutes procyonoides*), the leopard cat (*Prionailurus bengalensis*), and the Eurasian otter (*Lutra lutra*). A total of 5 host-specific parasite species were identified, including 2 from raccoon dogs, 2 from leopard cats, and 1 from Eurasian otters. In addition, numerous parasite species of their prey animals were detected in their feces. It was found that the parasitome composition varied between host animals, and it was thought that the difference was attributed to the difference in prey animals, as numerous small mammal parasites were detected from feces of leopard cats inhabiting inland areas and fish parasites from feces of Eurasian otters and raccoon dogs inhabiting waterside areas. Furthermore, 5 zoonotic parasites known to infect humans were identified at the species level. Wildlife-associated zoonoses are expected to increase as the proximity between humans and wildlife increases due to urbanization. Vigilance may be necessary, such as by monitoring parasites in wildlife feces, as was done in this study.

## Highlights

• Fecal parasitomes of raccoon dogs, leopard cats, and Eurasian otters investigated.
• The parasitomes varied between host animals, likely due to difference in prey.
• Zoonotic parasites including nematodes and flatworms identified.
• Vigilance and continuous monitoring for parasites in wildlife may be necessary.

## Graphical abstract

**Figure:**
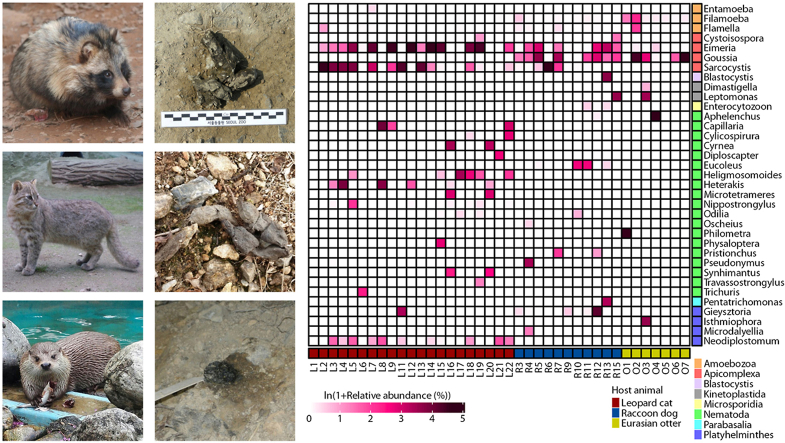

## 1 Introduction

Parasites are one of the most understudied pathogens, despite the fact that large numbers of people are at risk of adverse health effects from their infections (Hotez et al., 2009). Furthermore, in addition to their impacts on human health, they cause pathological conditions in livestock, hinder food intake and growth, and reduce livestock productivity (Sykes, 1994). Additionally, parasite infections are impacting the health of wildlife, including threatened species, raising concerns about their population declines (Pedersen et al., 2007). However, previous research on parasites has mainly focused on those that pose a high risk to humans and livestock (Thompson et al., 2010), and their diversity, host relationships, and ecosystem roles are still poorly understood (Thompson et al., 2010; Carlson et al., 2020). For example, there are an estimated 100,000–350,000 species of endoparasites in vertebrates, of which 85%–95% are still scientifically undescribed (Carlson et al., 2020). In particular, as urbanization increases the risk of contact between humans and wildlife (Hassell et al., 2017), there is a growing need for research on zoonotic parasites in wildlife from the perspective of One Health.

Parasite identification has historically been based primarily on morphological observations, and more recently by serological, molecular, and proteomics-based methods (Ndao, 2009). For example, morphological identification is conducted by observing the egg, cyst, or larval morphology unique to each parasite (Aivelo and Medlar, 2018). However, these methods have drawbacks, such as the need for specialized knowledge of parasite morphology, the need for laborious observation, and the inability to objectively identify parasite species. On the other hand, among the newly adopted methods, the method using DNA barcodes has the advantages of being able to identify the species of parasites with high sensitivity and objectiveness, and to analyze their composition and diversity. As examples of DNA barcode markers for parasites, the mitochondrial cytochrome oxidase subunit Ⅰ (Moszczynska et al., 2009), the 18S ribosomal RNA (rRNA) gene (Tanaka et al., 2014), and the internal transcribed spacer 2 region (Avramenko et al., 2015) have been used. Recently, a method using multiple primer pairs targeting the 18S rRNA genes of a wide variety of parasites has also been introduced, enabling comprehensive characterization of the parasites in environmental samples (Cannon et al., 2018).

Here, we aimed to characterize the diversity of parasitomes (collections of parasites) in feces of three species of wild carnivores inhabiting Korea, namely, the raccoon dog (*Nyctereutes procyonoides*), the leopard cat (*Prionailurus bengalensis*), and the Eurasian otter (*Lutra lutra*), including zoonotic parasites and parasites of their prey animals. In South Korea, the raccoon dog maintains decent population, but the leopard cat and the Eurasian otter are classified as endangered species (National Institute of Biological Resources, 2014). To comprehensively detect species of parasites in carnivorous wild animals in Korea, we used the method reported by Cannon et al. (2018), in which 13 primer pairs are used to amplify 18S rRNA genes of parasites belonging to nine different taxonomic groups: Amoebozoa, Apicomplexa, *Blastocystis*, Diplomonadida, Kinetoplastida, Microsporidia, Nematoda, Parabasalia, and Platyhelminthes. To our knowledge, few studies have investigated the parasitomes of those carnivorous wildlife in Korea.

## 2 Materials and methods

### 2.1 Fecal samples

Fecal samples previously collected for diet studies of the wild animals (Kumari et al., 2019; Woo et al., 2022, 2023) were used. A total of 40 fecal samples were collected, consisting of 11, 22, and 7 samples of raccoon dogs, leopard cats, and Eurasian otters, respectively. Sample metadata are listed in Supplementary Table 1 and sample collection methods are reported in our previous studies (Kumari et al., 2019; Woo et al., 2022, 2023). Briefly, fecal samples of raccoon dogs and Eurasian otters were collected from a waterside area (Woo et al., 2022) and an estuary area (Kumari et al., 2019), respectively. Fecal samples of leopard cats were collected in inland areas (Woo et al., 2023). Approximately 10 g of each sample was collected with a sterile wooden spatula in the field. Each collected sample was placed in a 50 ml conical tube and transported to the laboratory with ice packs. In our previous studies (Kumari et al., 2019, 2021), the host of each fecal sample was confirmed by PCR specific for each animal. The transported samples were stored at −80 °C until analysis.

### 2.2 DNA extraction

DNA was extracted from the collected fecal samples using a PowerMax® Soil DNA Isolation Kit (Mobio Laboratory, Inc., Carlsbad, CA, USA). Prior to DNA extraction, each sample was manually homogenized using 5 ml of ultra-pure water and a sterilized wooden spatula. Approximately 0.2 g of each homogenized sample was introduced into a 2 ml tube supplied with the kit. To enhance DNA extraction efficiency, 300 mg of 0.1 mm diameter glass bead and 100 mg of 0.5 mm diameter glass bead were added to the kit’s 2 ml tube. Each sample was homogenized for 3 min using a bead beater (BioSpec Products, Inc., Bartlesville, OK, USA). Then, DNA was purified and eluted in 50 μl of Tris-EDTA buffer according to the kit's protocol. The extracted DNA was stored at −80 °C until analysis.

### 2.3 DNA sequencing

The method reported by Cannon et al. (2018) was used. Briefly, for each sample, 13 different primer pairs were used to amplify the 18S rRNA genes of nine different groups of parasites: Amoebozoa, Apicomplexa, *Blastocystis*, Diplomonadida, Kinetoplastida, Microsporidia, Nematoda, Parabasalia, and Platyhelminthes. PCR primers and reaction conditions were according to the method reported by Cannon et al. (2018). These PCR assays used primers with the adapter sequences of Illumina MiSeq (Illumina, Inc., San Diego, CA, USA). PCR products were checked on an agarose gel with SYBR™ Gold Nucleic Acid Gel Stain (Thermo Fisher Scientific, Inc., Waltham, MA, USA) and those with unintended lengths were excluded. PCR products amplified with 13 different primer pairs derived from each fecal sample were purified by AMPure XP beads (Beckman Coulter, Inc., Brea, CA, USA). The purified DNA samples were quantified using a DS-11 FX (DeNovix, Inc., Wilmington, DE, USA), normalized to the lowest concentration among the samples, and diluted with 10 mM Tris pH 8.5. The normalized DNA samples from each fecal sample were pooled into a single sample. Each pooled sample was tagged by index PCR using a Nextera XT Index Kit v2 (Illumina). After index PCR, the products were purified with AMPure XP beads (Beckman Coulter). The purified DNA samples were quantified using a Quant-iT PicoGreen dsDNA reagent kit (Life Technologies, Carlsbad, CA, USA). The samples were diluted with 10 mM Tris pH 8.5 and normalized to 4 nM. The normalized DNA samples were loaded onto a v3 600 cycle-kit reagent cartridge (Illumina) with 30% PhiX internal control for 2 × 300 bp paired-end sequencing on an Illumina MiSeq system. Negative controls were included in all PCRs and had no amplification.

### 2.4 Data processing and analysis

Trimming adapter and index sequences and removing reads with a quality score <20 were performed using MiSeq Reporter version 2.5 (Illumina). Additionally, low-quality reads with >1.0 expected errors and reads with less than 150 bp in length were removed using USEARCH version 11.0.667 (Edgar, 2010). Then, the UNOISE algorithm (Edgar and Flyvbjerg, 2015) was used to identify zero-radius operational taxonomic units (ZOTUs) and remove chimeric reads. Additionally, if the forward and reverse primer sequence pairs contained in the ZOTU sequences were from PCRs used to detect different parasite groups, they were determined to be chimeric and manually excluded. Each of resultant ZOTUs was taxonomically assigned by BLASTN version 2.14.0+ against the NCBI nucleotide database (nt) in May 2023. Taxonomic assignments were performed based on the lowest E-value, with cut-off values for E-value and alignment identity set at <1e^−10^ and >99%, respectively. For species-level identification, only cases where the top hit of each ZOTU was assigned to only one species were allowed, and all other cases were regarded ambiguous. If multiple species belonging to the same genus, family, order, or class were assigned as top hits, they were considered ambiguous at the species level and assigned to a taxon that was unambiguous at the taxonomic level higher than the species level. Unintended reads from non-parasites such as fungi, bacteria, and arthropods that might have been amplified due to the inadequate primer specificity (Cannon et al., 2018) were excluded. In addition, phylogenetic tree analysis was performed to confirm the BLASTN results. Specifically, the sequences of the top 100 BLASTN hits of each ZOTU were aligned with those of the ZOTUs by the MUSCLE (Edgar, 2004) and the phylogenetic tree was constructed with the neighbor-joining method (Saitou and Nei, 1987) for each parasite species.

### 2.5 Statistical analysis

Statistical analysis was performed using the phyloseq package (McMurdie and Holmes, 2013) and vegan package (Oksanen et al., 2017) on R version 4.1.0. The diversity of parasites within (α diversity) and between (β diversity) samples were analyzed. For α diversity, the Chao1 richness estimator and Shannon index were calculated to characterize parasite richness and diversity within each sample, respectively. Kruskal–Wallis and *post hoc* Wilcoxon rank-sum tests were performed to compare these α diversity metrics between the host animals. For β diversity, the Jaccard index and Bray–Curtis dissimilarity were calculated to characterize differences in parasite membership and structure between samples, respectively. Permutational multivariate analysis of variance (PERMANOVA) was performed to compare differences in parasite membership and structure between the host animals.

### 2.6 Data availability

Raw sequence data are available at NCBI under the BioProject number PRJNA934160.

## 3 Results

### 3.1 Sequencing statistics

A total of 2,093,268 high-quality sequence reads were generated from a total of 39 fecal samples (Supplementary Table 2). One of the leopard cat samples (L10) was excluded because the target organisms were not amplified. Of these sequence reads obtained, approximately 43.6% (912,196 reads) were assigned to the target parasites. The remaining reads were excluded from subsequent analyses, either because they were not classified into the targeted parasites or did not meet criteria of E-value and/or alignment identity.

### 3.2 Diversity

**Fig. 1:**
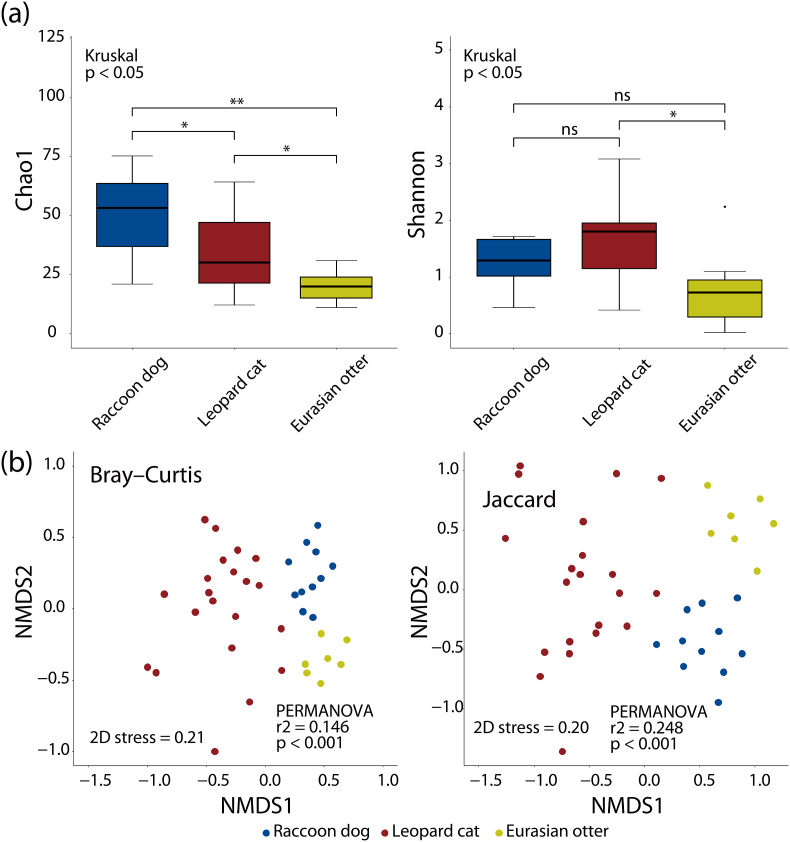
Diversity of fecal parasitomes of wild carnivores in Korea. The results shown are based on the diversity of zero-radius operational taxonomic units (ZOTUs) that were taxonomically assigned to parasites. (a) Comparison of richness and diversity of parasite ZOTUs between host animals estimated by the Chao1 estimator and Shannon index, respectively. (b) Non-metric multidimensional scaling (NMDS) plots showing the structure and membership of parasite ZOTUs represented by the Bray–Curtis dissimilarity and Jaccard index, respectively. In the panel (a), one asterisk (*) and two asterisks (**) represent *p* < 0.05 and *p* < 0.01, respectively, by the *post hoc* Wilcoxon rank-sum test. The abbreviation “ns” represents no statistical difference.

Statistical differences were found in the Chao1 estimator and Shannon index, which represent within-sample diversity (α diversity) of parasite ZOTUs, between the host animals (*p* < 0.05; Kruskal Wallis rank sum test) (Fig. 1a), indicating that the parasite richness in the samples are different between the host animals. Similarly, differences were identified in between-sample diversity (β diversity), such as structure (Bray–Curtis dissimilarity) and membership (Jaccard index) of parasite ZOTUs, between the host animals (*r*^2^ = 0.146 and 0.248, respectively, *p* < 0.001; PERMANOVA) (Fig. 1b), indicating that the parasite species that constitute the parasitome are different for each host animal.

### 3.3 Taxonomic composition

**Fig. 2:**
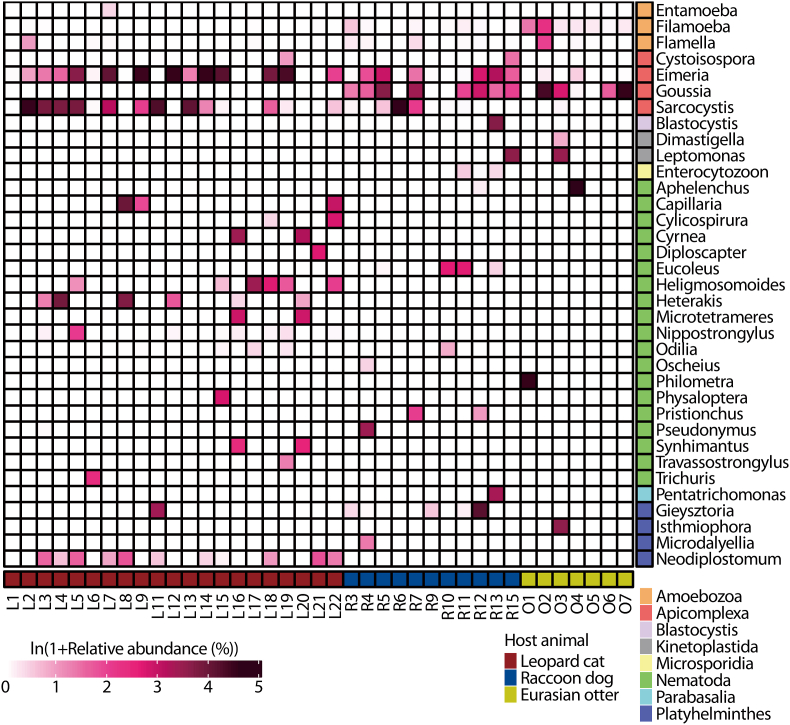
Relative abundance of all parasite genera detected from fecal samples of wild carnivores in Korea. The relative abundance of each parasite is defined as the ratio of the number of sequence reads assigned to that parasite to the total number of sequence reads assigned to all target parasites.

Numerous parasites belonging to eight target groups, excluding Diplomonadida, were detected (Fig. 2). A total of 35 parasite genera, including parasites of prey animals of the carnivorous animals investigated in this study, were identified, i.e., 18 from raccoon dogs, 22 from leopard cats, and 11 from Eurasian otters (Supplementary Table 3). Among Apicomplexa genera, *Goussia*, *Eimeria*, and *Sarcocystis* were abundantly detected. In particular, *Goussia* was detected in large amounts in the feces of Eurasian otters and raccoon dogs. *Eimeria* and *Sarcocystis* were abundantly detected in the feces of leopard cats. Among Nematoda genera, *Heterakis* was abundantly detected in the feces of leopard cats. In addition, *Philometra* was detected in a large amount in a fecal sample of the Eurasian otter. Among Platyhelminthes genera, *Neodiplostomum* was abundantly detected in fecal samples of leopard cats. Information on parasites identified at the species level and their known hosts is provided in Supplementary Tables 4 and 5

### 3.4 Zoonotic parasites

Some of the detected DNA had sequences identical or highly similar to 18S rRNA gene sequences of zoonotic parasites known to parasitize humans. Table 1 shows 5 zoonotic parasite species with sequences identical or highly similar to those of ZOTUs detected in this study. The five species are *Enterocytozoon bieneusi*, *Capillaria hepatica*, *Trichuris vulpis*, *Pentatrichomonas hominis*, and *Isthmiophora hortensis*, and ZOTUs assigned to those species were unambiguously assigned only as those species without being top-hit with other species at the same E-value. Their phylogenetic trees are shown in Supplementary Figs. 1–5, and information on the DNA sequences of ZOTUs assigned to these zoonotic parasites is provided in Supplementary Data 1.

**Table 1:** Zoonotic parasites detected from fecal samples of wild carnivores in Korea. Phylogenetic trees and sequences of ZOTUs assigned to these zoonotic parasites are provided in Supplementary Figs. 1–5 and Supplementary Data 1, respectively.

Group	Species	Number of samples detected			Definitive and/or intermediate hosts	Reference(s)
		Raccoon dog (n = 11)	Leopard cat (n = 21)	Eurasian otter (n = 7)		
Microsporidia	*Enterocytozoon bieneusi*	3	n.d.	n.d.	Primates, pigs, cattle, horses, llamas, kudus, dogs, cats, foxes, raccoon, otters, guinea pig, beavers, rabbits, muskrats, falcons, and other birds	Santin and Fayer (2011)
Nematoda	*Capillaria hepatica*	n.d.	3	n.d.	Rodents and mammals	Fuehrer (2014a, 2014b)
	*Trichuris vulpis*	n.d.	1	n.d.	Canine	Dunn et al. (2002)
Parabasalia	*Pentatrichomonas hominis*	1	n.d.	n.d.	Mammals	Maritz et al. (2014)
Platyhelminthes	*Isthmiophora hortensis*	n.d.	n.d.	1	Rats, dogs, cats, freshwater snails, loaches, and freshwater fish	Chai and Jung (2019)

*Abbreviation:* n.d., not detected.

## 4 Discussion

In this study, we successfully characterized the fecal parasitomes of raccoon dogs, leopard cats, and Eurasian otters inhabiting Korea by 18S rRNA gene sequencing using multiple primer pairs. We found that the richness, structure, and membership of parasites varied greatly between the host animals. Furthermore, we confirmed the detection of DNA sequences identical or highly similar to the 18S rRNA gene sequences of 5 zoonotic parasites known to parasitize humans. The method used in this study has the advantage of being able to detect a wide range of parasite groups from a sample compared to the traditional methods. For example, the method we used can detect not only host-specific parasites but also non-host-specific parasites. Therefore, a comprehensive understanding of parasites can be achieved without being bound by *a priori* assumptions. Furthermore, sequencing, such as bacterial 16S rRNA gene sequencing, generally proceeds to one region as a target and uses a single primer pair, but the method of pooling each PCR product with multiple primer pairs has an advantage in terms of sequencing cost.

Differences in parasitomes between the host animals are most likely due to their dietary content. For example, genera containing fish parasites, such as the apicomplexa *Goussia* (Dyková and Lom, 1981) and the nematode *Philometra* (Nguyen et al., 2021), were abundantly detected in the feces of raccoon dogs and/or Eurasian otter. Our dietary research revealed that fish is the main food of raccoon dogs inhabiting the waterside area (Woo et al., 2022) and Eurasian otters inhabiting the estuary area (Kumari et al., 2019) in Korea. In addition, in the feces of leopard cats, genera containing species whose intermediate and/or definitive hosts are small mammals such as rodents and/or birds, e.g., the apicomplexa *Eimeria* (Todd and Lepp, 1971) and *Sarcocystis* (Prakas et al., 2019), the nematode and *Heterakis* (Smith, 1953; Katherine Lynn and Robert Byron, 2019), and the platyhelminth *Neodiplostomum* (Sanmartin et al., 2004), were abundantly detected. Our dietary study revealed that murids and birds are the main prey of leopard cats in the inland areas (Woo et al., 2023). It is well known that parasites pass from prey to predators, in which they are digested to death or an infection is established (Thieltges et al., 2013). We consider that the observed differences in parasitomes are due to each host preying on different animals and thus concomitantly taking up different species of parasites.

We also detected DNA with sequences identical or highly similar to those of zoonotic parasites that can parasitize humans. For example, DNA with sequences highly similar to those of the microsporidia *Enterocytozoon bieneusi* was detected in some fecal samples of raccoon dogs. The hosts of *E. bieneusi* are wide-ranging, including canids, felids, and primates including humans (Santin and Fayer, 2011). It has been reported that the DNA of *E. bieneusi* was detected in the feces of raccoon dogs in Korea (Amer et al., 2019). DNA with a sequence identical to those of the parabasalid *Pentatrichomonas hominis* was also detected from a fecal sample of raccoon dog. *P. hominis* is considered zoonotic and is known to parasitize mammals such as humans, monkeys, cats, dogs, and rats (Maritz et al., 2014). It has been reported that *P. hominis* was detected in farmed raccoon dogs in China (Li et al., 2017).

*Capillaria hepatica*, a zoonotic nematode, was detected in some leopard cat feces. *C. hepatica* is known to have a wide range of hosts including rodents (Fuehrer, 2014a) and other mammals, such as domestic cats (Fuehrer, 2014b). There have been no reports that *C. hepatica* has been detected in the leopard cat, but it has been reported that it was detected in the Sunda leopard cat (*Prionailurus javanensis*), a close relative of the leopard cat, in the Philippines (Lo et al., 2021). A zoonotic nematode *Trichuris vulpis*, which is known to have canids as their definitive hosts (Dunn et al., 2002), was also detected from a fecal sample of the leopard cat. Few cases of the detection of *T. vulpis* from felines have been reported, and we do not know the reason. One hypothesis is that the leopard cat concomitantly preyed on the dog parasitized with *T. vulpis*, because our dietary study identified the dog as a dietary item of leopard cats in our study areas (Woo et al., 2023).

From a fecal sample of Eurasian otter, four ZOTUs with DNA sequences similar to the DNA sequence of the platyhelminth *Isthmiophora hortensis* (Supplementary Data 1), but well-separated phylogenetically (Supplementary Fig. 5), were detected. These ZOTUs may be derived from a novel species of the *Isthmiophora* genus, but further studies are needed to prove this. The detection of *Isthmiophora* species seems reasonable because the *Isthmiophora* genus includes fish parasites (Chai and Jung, 2019). In fact, it has been reported that several species of *Isthmiophora* have been detected in carcasses of Eurasian otters, such as *Isthmiophora inermis* in Korea (Choe et al., 2019) and *Isthmiophora melis* in Denmark (Takeuchi-Storm et al., 2021). Additionally, the *Isthmiophora* genus, including *I. hortensis*, has been reported to be prevalent in wildlife, especially mustelids, in Korea (Choe et al., 2019).

Finally, we note that analysis of sequencing data, especially removal of chimeras, requires careful attention. Some of our ZOTUs consisted of sequences from different pairs of forward and reverse primers, which we manually removed. In addition, we also note that sequencing that includes the PCR process cannot be free from PCR bias, and in particular, PCR bias can make it difficult to detect taxa that exist in small quantities (Gonzalez et al., 2012; Cannon et al., 2018).

## 5 Conclusion

In Korea, an increase in wildlife-associated diseases has been reported in recent decades (Hwang et al., 2017). Therefore, it is imperative to monitor zoonotic pathogens, including zoonotic parasites, harbored by wildlife. In this study, we investigated the diversity of parasitomes, including zoonotic parasites, associated with the carnivorous wildlife in Korea by using 18S rRNA gene sequencing with multiple primer pairs. We found that the parasitome richness, structure, and membership differed between the host animals, and that the detected zoonotic parasites were plausible in light of the animal species known to be their hosts, as well as the predatory propensities of the host animals. In the future, it is expected that zoonotic diseases will further increase due to the increased proximity between humans and wildlife due to urbanization (Hassell et al., 2017). Vigilance may need to be continuously exercised, such as by monitoring parasites in the feces of wildlife, as was done in this study.

## Funding

This work was supported by 10.13039/501100002551Seoul National University Research Grant in 2018 (W-SL, JK and NY) and by the General Researcher Program of the 10.13039/501100003725National Research Foundation of Korea (2021R1F1A1060259) (NY).

## Declaration of competing interest

None to declare.
