# Supplementary material for: Diversity of fecal parasitomes of wild carnivores inhabiting Korea, including zoonotic parasites and parasites of their prey animals, as revealed by 18S rRNA gene sequencing
Source: Int J Parasitol Parasites Wildl. 2023 Jun 3;21:179–84. doi: 10.1016/j.ijppaw.2023.05.005 (PMC10267430; doi:10.1016/j.ijppaw.2023.05.005)
Supplement: Multimedia component 1 [file mmc1.pdf]

Supporting Information for:

Diversity of fecal parasitomes of wild carnivores inhabiting Korea, including zoonotic parasites and parasites of their prey animals, as revealed by 18S rRNA gene sequencing

Cheolwoon Woo [a], Mohammad Imtiaj Uddin Bhuiyan [a], Kyung Yeon Eo [b], Woo-Shin Lee [c], Junpei Kimura [d] and Naomichi Yamamoto [a,e*]

[a.] Department of Environmental Health Sciences, Graduate School of Public Health, Seoul National University, Seoul 08826, Republic of Korea

[b.] Department of Animal Health and Welfare, College of Healthcare and Biotechnology, Semyung University, Jecheon 27136, Republic of Korea

[c.] Department of Forest Sciences, College of Agriculture and Life Science, Seoul National University, Seoul 08826, Republic of Korea

[d.] College of Veterinary Medicine, Seoul National University, Seoul 08826, Republic of Korea

[e.] Institute of Health and Environment, Graduate School of Public Health, Seoul National University, Seoul 08826, Republic of Korea

[*]Correspondence: E-mail: nyamamoto@snu.ac.kr (NY)

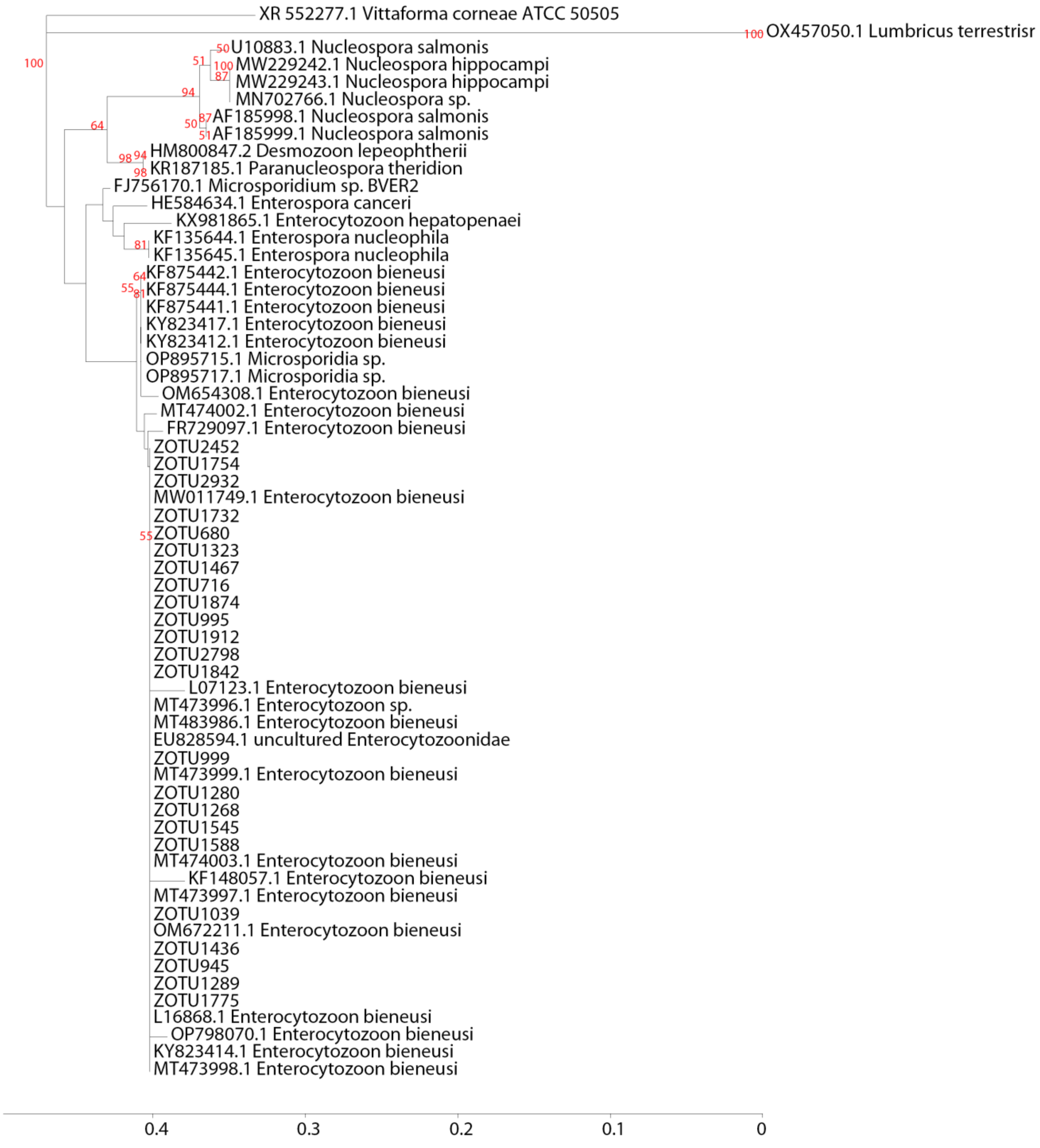

Supplementary Fig. 1. Phylogenetic tree of the 23 ZOTUs taxonomically assigned as *Enterocytozoon bieneusi* and the top 100 BLASTN hits of the ZOTUs. Aligned sequences to ZOTUs with 100% similarity were merged, and multiple sequence alignment was performed using the MUSCLE (Edgar, 2004). Phylogenetic tree construction was performed based on the neighbor-joining method (Saitou and Nei, 1987) on the ape package (Paradis et al., 2004) of R version 4.1.0. The values in red represent percentages of bootstrap values based on 1000 resamplings. The sequence of *Vittaforma corneae* is used as the root.

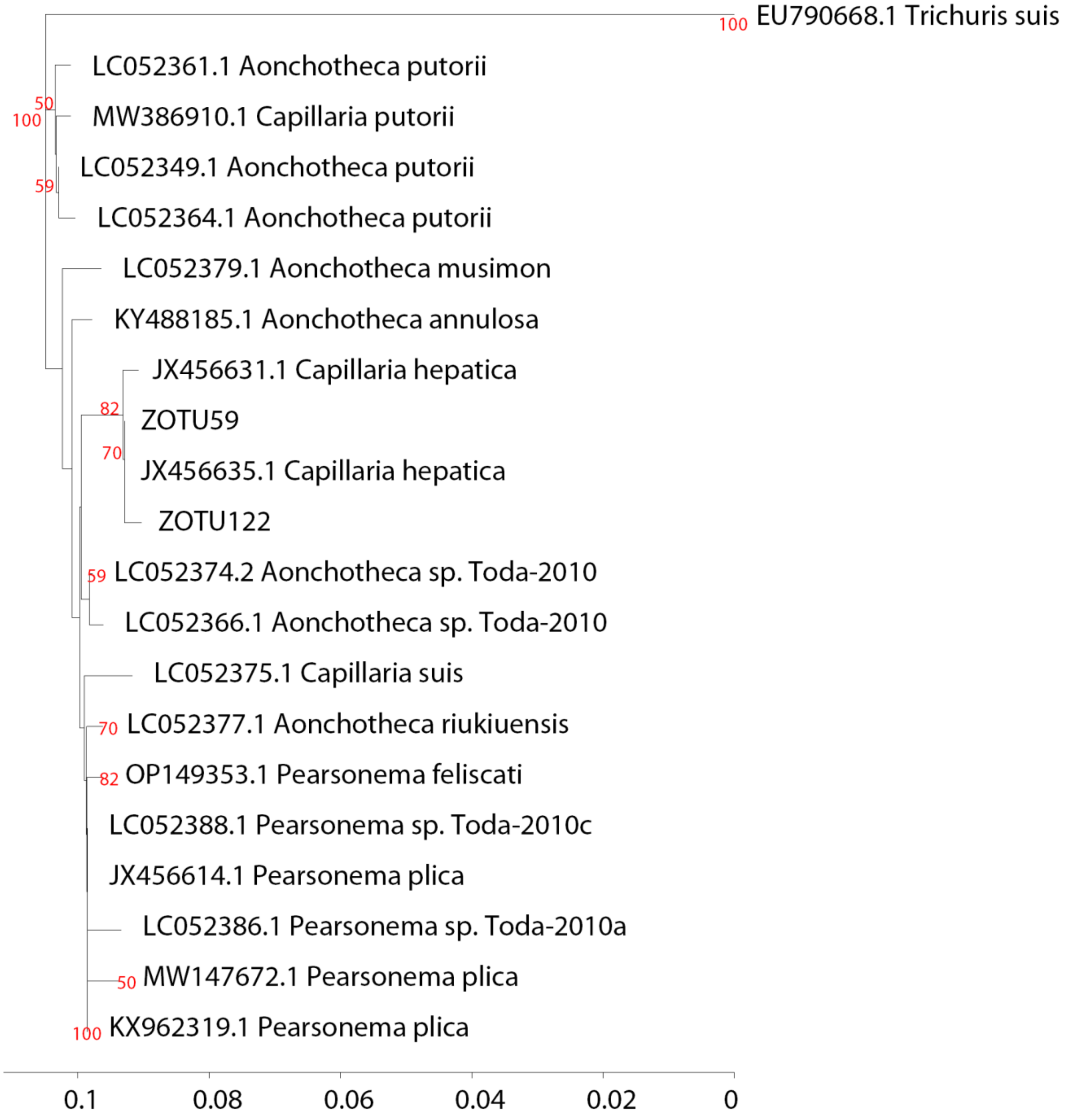

Supplementary Fig. 2. Phylogenetic tree of the 2 ZOTUs taxonomically assigned as *Capillaria hepatica* and the top 100 BLASTN hits of the ZOTUs. Aligned sequences to ZOTUs with 100% similarity were merged, and multiple sequence alignment was performed using the MUSCLE (Edgar, 2004). Phylogenetic tree construction was performed based on the neighbor-joining method (Saitou and Nei, 1987) on the ape package (Paradis et al., 2004) of R version 4.1.0. The values in red represent percentages of bootstrap values based on 1000 resamplings. The sequence of *Trichuris suis* is used as the root.

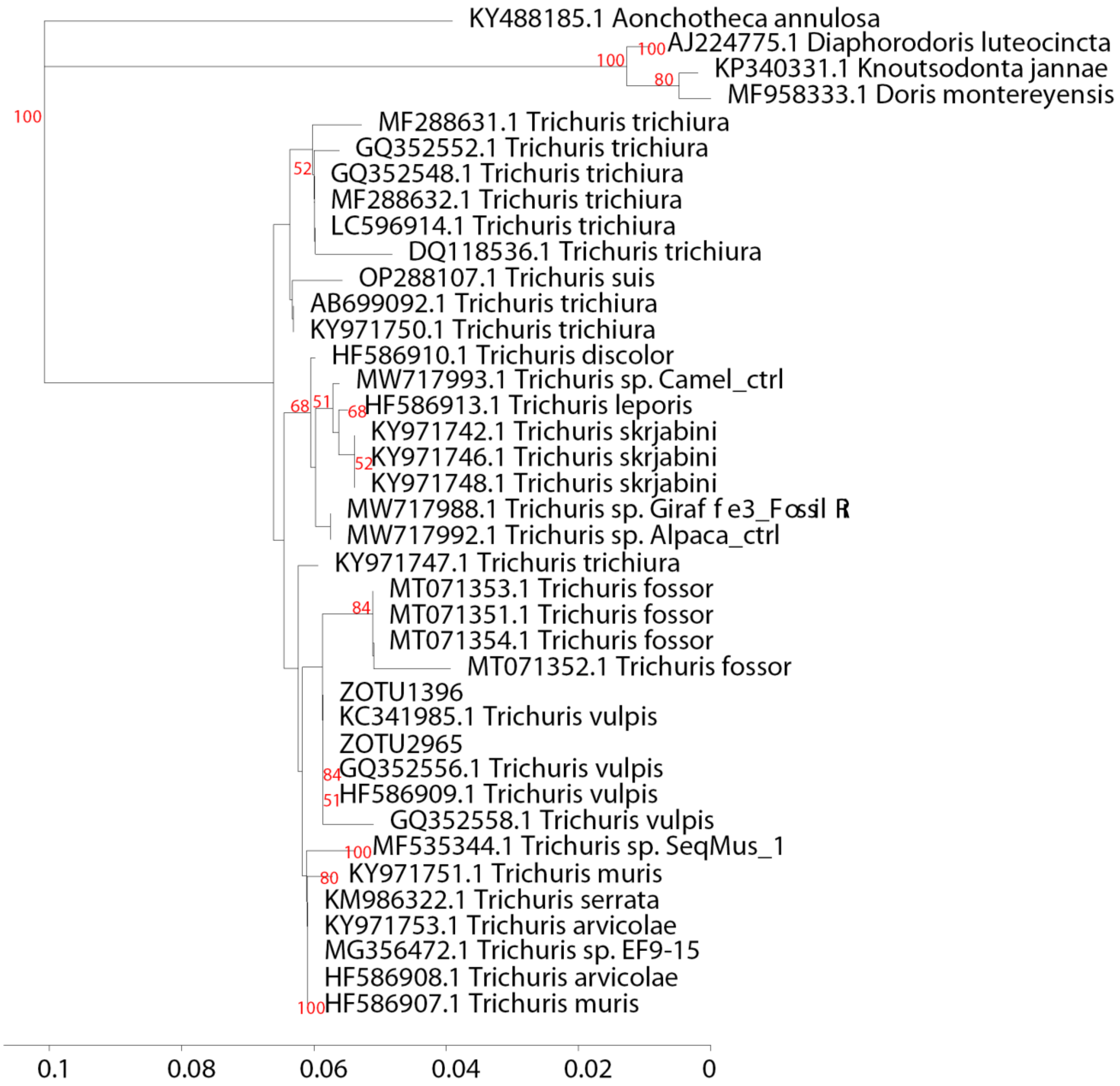

Supplementary Fig. 3. Phylogenetic tree of the 2 ZOTUs taxonomically assigned as *Trichuris vulpis* and the top 100 BLASTN hits of the ZOTUs. Aligned sequences to ZOTUs with 100% similarity were merged, and multiple sequence alignment was performed using the MUSCLE (Edgar, 2004). Phylogenetic tree construction was performed based on the neighbor-joining method (Saitou and Nei, 1987) on the ape package (Paradis et al., 2004) of R version 4.1.0. The values in red represent percentages of bootstrap values based on 1000 resamplings. The sequence of *Aonchotheca annulosa* is used as the root.

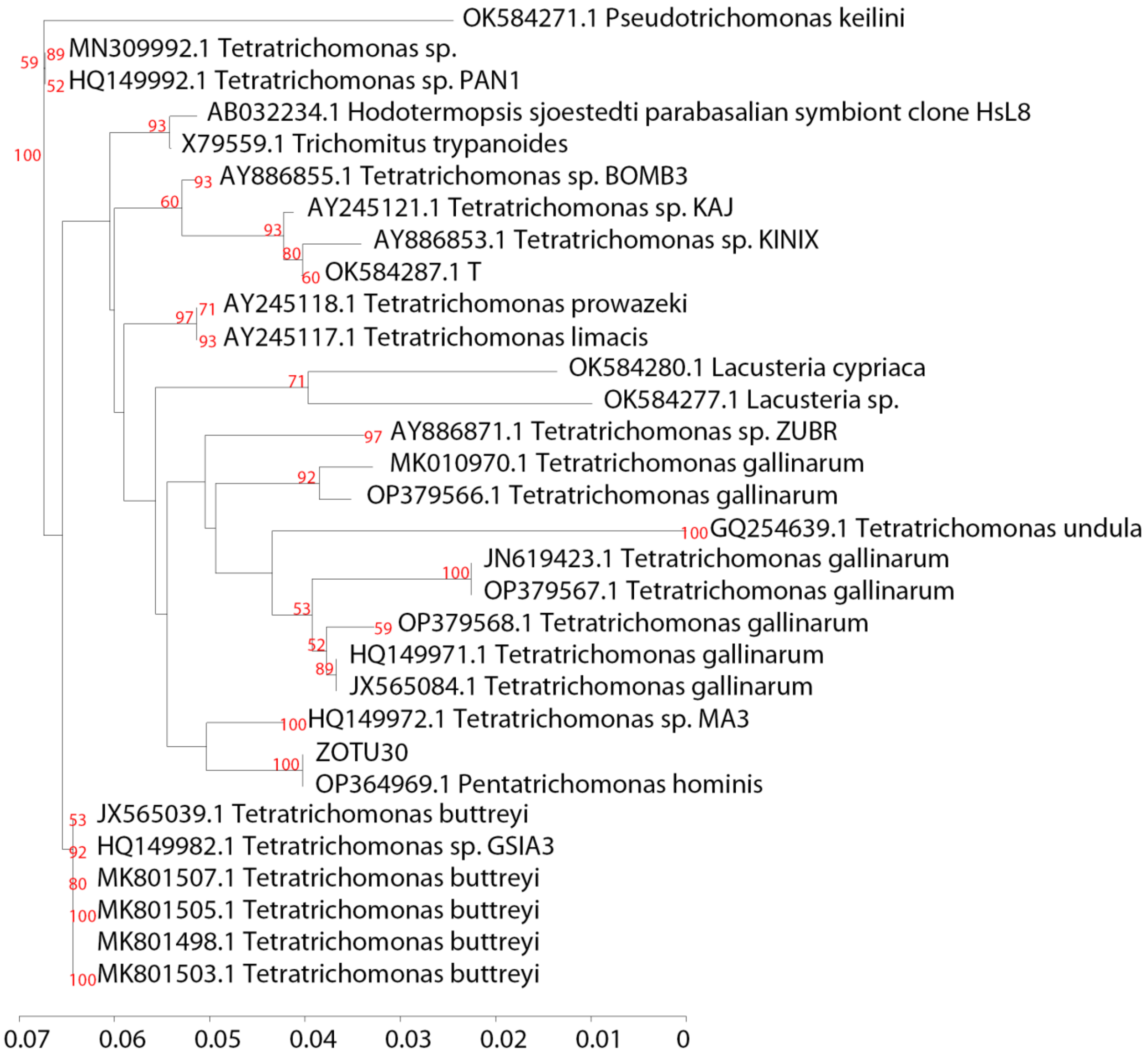

Supplementary Fig. 4. Phylogenetic tree of the ZOTU taxonomically assigned as *Pentatrichomonas hominis* and the top 100 BLASTN hits of the ZOTU. Aligned sequences to ZOTU with 100% similarity were merged, and multiple sequence alignment was performed using the MUSCLE (Edgar, 2004). Phylogenetic tree construction was performed based on the neighbor-joining method (Saitou and Nei, 1987) on the ape package (Paradis et al., 2004) of R version 4.1.0. The values in red represent percentages of bootstrap values based on 1000 resamplings. The sequence of *Pseudotrichomonas keilini* is used as the root.

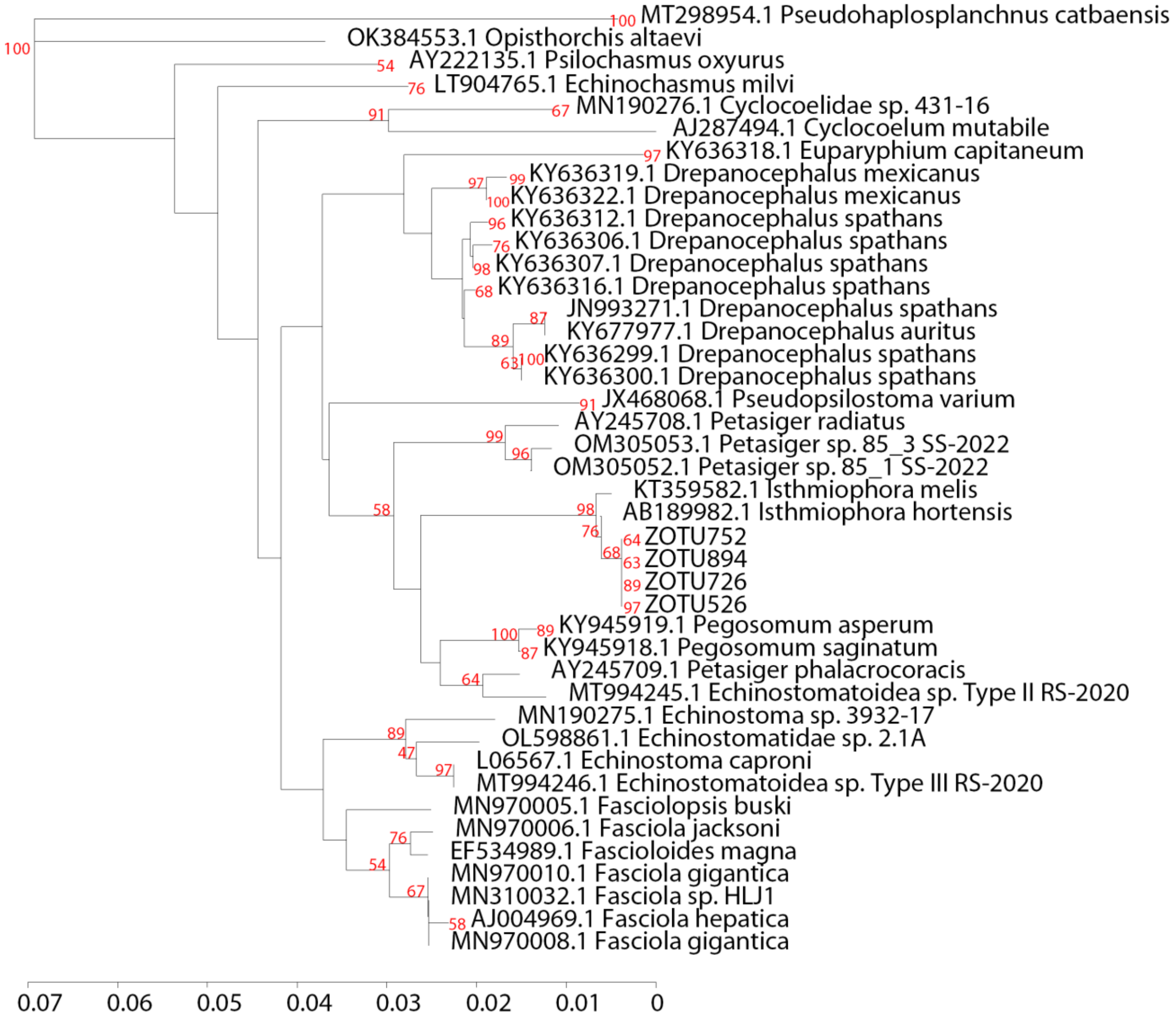

Supplementary Fig. 5. Phylogenetic tree of the 4 ZOTUs taxonomically assigned as *Isthmiophora hortensis* and the top 100 BLASTN hits of the ZOTUs. Aligned sequences to ZOTUs with 100% similarity were merged, and multiple sequence alignment was performed using the MUSCLE (Edgar, 2004). Phylogenetic tree construction was performed based on the neighbor-joining method (Saitou and Nei, 1987) on the ape package (Paradis et al., 2004) of R version 4.1.0. The values in red represent percentages of bootstrap values based on 1000 resamplings. The sequence of *Pseudohaplosplanchnus catbaensis* is used as the root.

Supplementary Table 1. Sample metadata.

Sample ID	Sampling date (YYYY/MM/DD)	Latitude	Longitude	City or Gun
R3	2017/05/21	36°40'34.80"N	126°28'10.85"E	Seosan, Chungcheongnam-do
R4	2017/05/21	36°40'07.82"N	126°28'40.65"E	Seosan, Chungcheongnam-do
R5	2017/05/21	36°39'59.61"N	126°28'46.67"E	Seosan, Chungcheongnam-do
R6	2017/05/21	36°40'18.71"N	126°29'13.25"E	Seosan, Chungcheongnam-do
R7	2017/05/21	36°40'15.01"N	126°30'25.57"E	Seosan, Chungcheongnam-do
R9	2017/05/21	36°39'39.54"N	126°27'49.62"E	Seosan, Chungcheongnam-do
R10	2017/05/21	36°39'41.34"N	126°27'55.72"E	Seosan, Chungcheongnam-do
R11	2017/05/21	36°39'45.32"N	126°28'08.19"E	Seosan, Chungcheongnam-do
R12	2017/05/21	36°39'47.84"N	126°28'16.84"E	Seosan, Chungcheongnam-do
R13	2017/05/21	36°37'50.97"N	126°29'28.88"E	Seosan, Chungcheongnam-do
R15	2017/05/21	36°37'44.85"N	126°28'37.22"E	Seosan, Chungcheongnam-do
L1	2019/02/20	36°36'1.83"N	127°8'2.71"E	Gongju, Chungcheongnam-do
L2	2019/02/20	36°34'32.91"N	127°9'43.39"E	Gongju, Chungcheongnam-do
L3	2019/02/21	36°27'19.5"N	127°9'1.1"E	Gongju, Chungcheongnam-do
L4	2019/02/21	36°27'18.28"N	127°9'2.04"E	Gongju, Chungcheongnam-do
L5	2019/02/21	36°27'17.45"N	127°9'1.79"E	Gongju, Chungcheongnam-do
L6	2019/02/21	36°27'11.21"N	127°8'55.71"E	Gongju, Chungcheongnam-do
L7	2019/02/21	36°30'52.96"N	127°19'41.42"E	Yeongi-gun, Chungcheongnam-do
L8	2019/02/20	36°36'10.53"N	127°7'38.15"E	Gongju, Chungcheongnam-do
L9	2019/02/20	36°36'4.58"N	127°7'54.92"E	Gongju, Chungcheongnam-do
L10	2019/02/20	36°36'4.8"N	127°7'53.11"E	Gongju, Chungcheongnam-do
L11	2019/02/20	36°35'43.81"N	127°8'47.17"E	Gongju, Chungcheongnam-do
L12	2019/02/20	36°35'44.95"N	127°8'46.55"E	Gongju, Chungcheongnam-do
L13	2019/02/21	36°27'17.87"N	127°8'58.7"E	Gongju, Chungcheongnam-do
L14	2019/02/21	36°30'59.4"N	127°19'38.73"E	Yeongi-gun, Chungcheongnam-do
L15	2019/02/05	36°17'55.8"N	128°7'27.12"E	Sangju, Gyeongsangbuk-do
L16	2019/02/20	35°45'13.2"N	128°23'44.52"E	Dalseong-gun, Daegu
L17	2019/02/20	35°44'54"N	128°23'42.72"E	Dalseong-gun, Daegu
L18	2019/02/20	35°44'17.64"N	128°24'3.42"E	Goryeong, Gyeongsangbuk-do
L19	2019/02/20	35°44'18"N	128°24'3.12"E	Goryeong, Gyeongsangbuk-do
L20	2019/02/20	35°44'17.46"N	128°24'3.66"E	Goryeong, Gyeongsangbuk-do
L21	2019/02/20	35°42'52.5"N	128°25'54.36"E	Goryeong, Gyeongsangbuk-do
L22	2019/02/20	35°42'13.8"N	128°23'39.9"E	Goryeong, Gyeongsangbuk-do
O1	2017/06/06	37°16'14.43"N	126°50'22.48"E	Ansan, Gyeonggi-do
O2	2017/06/06	37°16'14.83"N	126°50'22.78"E	Ansan, Gyeonggi-do
O3	2017/06/06	37°16'16.26"N	126°50'25.74"E	Ansan, Gyeonggi-do
O4	2017/06/06	37°16'14.82"N	126°50'27.78"E	Ansan, Gyeonggi-do
O5	2017/06/06	37°16'14.97"N	126°50'31.24"E	Ansan, Gyeonggi-do
O6	2017/06/06	37°16'14.23"N	126°50'31.32"E	Ansan, Gyeonggi-do
O7	2017/06/06	37°16'22.62"N	126°50'24.70"E	Ansan, Gyeonggi-do

Supplementary Table 2. DNA sequencing statistics.

Sample ID	Accession number	No. reads assigned to targeted organisms [a]	No. reads assigned to non-targeted organisms	Total
R3	SAMN33268131	4,382	49,833	54,215
R4	SAMN33268132	18,191	30,243	48,434
R5	SAMN33268133	23,724	29,979	53,703
R6	SAMN33268134	59,077	14,359	73,436
R7	SAMN33268135	2,691	47,266	49,957
R9	SAMN33268136	3,920	34,519	38,439
R10	SAMN33268137	6,718	23,117	29,835
R11	SAMN33268138	3,663	36,037	39,700
R12	SAMN33268139	30,380	37,578	67,958
R13	SAMN33268140	44,376	17,394	61,770
R15	SAMN33268141	27,547	44,980	72,527
L1	SAMN33268142	9,450	48,104	57,554
L2	SAMN33268143	11,365	52,076	63,441
L3	SAMN33268144	28,694	22,802	51,496
L4	SAMN33268145	37,452	31,361	68,813
L5	SAMN33268146	24,802	43,453	68,255
L6	SAMN33268147	1,941	32,293	34,234
L7	SAMN33268148	33,651	26,997	60,648
L8	SAMN33268149	11,284	25,246	36,530
L9	SAMN33268150	22,730	52,975	75,705
L11	SAMN33268151	25,448	37,701	63,149
L12	SAMN33268152	64,890	27,637	92,527
L13	SAMN33268153	38,356	32,502	70,858
L14	SAMN33268154	49,404	24,791	74,195
L15	SAMN33268155	38,200	19,310	57,510
L16	SAMN33268156	43,497	13,626	57,123
L17	SAMN33268157	17,862	18,369	36,231
L18	SAMN33268158	39,550	12,838	52,388
L19	SAMN33268159	46,837	14,341	61,178
L20	SAMN33268160	28,162	17,501	45,663
L21	SAMN33268161	23,494	22,836	46,330
L22	SAMN33268162	26,215	5,771	31,986
O1	SAMN33268163	9,351	37,045	46,396
O2	SAMN33268164	511	39,426	39,937
O3	SAMN33268165	7,264	27,706	34,970
O4	SAMN33268166	5,014	42,901	47,915
O5	SAMN33268167	18,806	23,461	42,267
O6	SAMN33268168	17,151	19,453	36,604
O7	SAMN33268169	6,146	43,245	49,391
Total		912,196	1,181,072	2,093,268

[a] Targeted organisms are Amoebozoa, Apicomplexa, *Blastocystis*, Diplomonadida, Kinetoplastida, Microsporidia, Nematoda, Parabasalia, and Platyhelminthes.

Supplementary Table 3. List of detected parasite genera and number of samples detected.

Group	Genus	Raccoon dog (n = 11)	Leopard cat (n = 21)	Eurasian otter (n = 7)
Amoebozoa	*Entamoeba*	n.d.	1	n.d.
	Filamoeba	10	9	7
	Flamella	8	8	3
Apicomplexa	*Cystoisospora*	1	2	n.d.
	Eimeria	10	20	5
	Goussia	8	n.d.	5
	Sarcocystis	9	15	1
Blastocystis	*Blastocystis*	1	n.d.	n.d.
Kinetoplastida	*Dimastigella*	n.d.	n.d.	1
	Leptomonas	1	n.d.	1
Microsporidia	*Enterocytozoon*	3	n.d.	n.d.
Nematoda	*Aphelenchus*	1	n.d.	1
	Capillaria	n.d.	3	n.d.
	Cylicospirura	n.d.	3	n.d.
	Cyrnea	n.d.	4	n.d.
	Diploscapter	n.d.	1	n.d.
	Eucoleus	4	n.d.	n.d.
	Heligmosomoides	n.d.	8	n.d.
	Heterakis	n.d.	7	n.d.
	Microtetrameres	n.d.	2	n.d.
	Nippostrongylus	n.d.	8	n.d.
	Odilia	1	6	n.d.
	Oscheius	1	n.d.	n.d.
	Philometra	n.d.	n.d.	1
	Physaloptera	n.d.	2	1
	Pristionchus	2	n.d.	n.d.
	Pseudonymus	1	2	n.d.
	Synhimantus	n.d.	2	n.d.
	Travassostrongylus	n.d.	2	n.d.
	Trichuris	n.d.	1	n.d.
Parabasalia	*Pentatrichomonas*	1	n.d.	n.d.
Platyhelminthes	*Gieysztoria*	8	5	n.d.
	Isthmiophora	n.d.	n.d.	1
	Microdalyellia	1	n.d.	n.d.
	Neodiplostomum	n.d.	11	n.d.

Abbreviation: n.d., not detected.

Supplementary Table 4. Parasite species detected from fecal samples of the wild carnivores in Korea that are known or likely to be host-specific for that animal.

Group	Species	Number of samples detected			Host specificity	Definitive and/or intermediate hosts, or sources of isolation	Reference(s)
		Raccoon dog (n = 11)	Leopard cat (n = 21)	Eurasian otter (n = 7)			
Microsporidia	*Enterocytozoon bieneusi*	3	n.d.	n.d.	Identified	Primates, pigs, cattle, horses, llamas, kudus, dogs, cats, foxes, raccoons, otters, guinea pigs, beavers, rabbits, muskrats, falcons, and other birds	Santin and Fayer (2011); Amer et al. (2019)
Nematoda	*Capillaria hepatica*	n.d.	3	n.d.	Identified in relative species	Rodents and mammals	Fuehrer (2014a, 2014b); Lo et al. (2021)
	Cylicospirura petrowi	n.d.	3	n.d.	Identified in relative species	Cats	Diakou et al. (2021)
Parabasalia	*Pentatrichomonas hominis*	1	n.d.	n.d.	Identified	Mammals	Maritz et al. (2014); Li et al. (2017)
Platyhelminthes	*Isthmiophora hortensis*	n.d.	n.d.	1	Possible, other species of the same genus have been detected	Rats, dogs, cats, freshwater snails, loaches, and freshwater fish	Chai and Jung (2019); Choe et al. (2019)

Abbreviation: n.d., not detected.

Supplementary Table 5. Parasite species detected from fecal samples of the wild carnivores in Korea that are not host-specific for that animal and likely from their prey animals.

Group	Species [a]	Number of samples detected			Definitive and/or intermediate hosts, or sources of isolation	Reference(s)
		Raccoon dog (n = 11)	Leopard cat (n = 21)	Eurasian otter (n = 7)		
Amoebozoa	*Entamoeba bovis*	n.d.	1	n.d.	Cattle, sheep, and reindeer	Stensvold et al. (2011)
Apicomplexa	*Eimeria adenoeides*	3	13	1	Turkeys	Augustine and Danforth (1990)
	Eimeria leucisci	5	3	n.d.	Cyprinid fish	Alvarez-Pellitero and Gonzalez-Lanza (1986); Rosenthal et al. (2016)
Kinetoplastida	*Dimastigella trypaniformis*	n.d.	n.d.	1	Isoptera	Hughes and Piontkivska (2003)
	Leptomonas jaculum	1	n.d.	1	*Nepa cinerea*	Lipa (1966)
Nematoda	*Aphelenchus avenae*	n.d.	n.d.	1	Plant-pathogenic fungi and *Agaricus bisporus*	Okada and Kadota (2003)
	Cyrnea leptoptera	n.d.	4	n.d.	*Milvus migrans* and *Falco tinnunculus*	Honisch and Krone (2008); Ebmer et al. (2020)
	Heligmosomoides thomomyos	n.d.	8	n.d.	Western pocket gophers	Hughes et al. (2021)
	Heterakis gallinarum	n.d.	2	n.d.	Gallinaceous birds	Katherine Lynn and Robert Byron (2019)
	Heterakis spumosa	n.d.	7	n.d.	Rats	Smith (1953)
	Microtetrameres cloacitectus	n.d.	2	n.d.	*Accipiter gentilis* and *A. nisus*	Honisch and Krone (2008)
	Nippostrongylus brasiliensis	n.d.	8	n.d.	Rats	Ogilvie and Jones (1971)
	Pseudonymus islamabadi	1	2	n.d.	Water beetles	Guzeeva and Spiridonov (2013)
	Synhimantus laticeps	n.d.	2	n.d.	Birds, terrestrial isopods, odonate, and dermapteran insects	Sanmartin et al. (2004)
	Trichuris vulpis	n.d.	1	n.d.	Canine	Dunn et al. (2002)
Platyhelminthes	*Neodiplostomum attenuatum*	n.d.	11	n.d.	Birds, amphibians, reptiles, and freshwater snails	Sanmartin et al. (2004)

[a] Species whose sources were environmental such as soil and water were excluded. In addition, free-living species, species for which host information has not been established, and species for which relevant literature was not found were excluded.
Abbreviation: n.d., not detected.

Supplementary Data 1. Information on sequence reads of ZOTUs assigned to zoonotic parasites.

Enterocytozoon bieneusi (Microsporidia)
>ZOTU680
GCAGGTTGATTCTGCCTGACGTAGATGCTAGTCTCTGAGATTAAGCCATGCATGTCAGTGAAGCCTT
ACGGCGGAACGGCGAACGGCTCAGTAATGTTGCGGTAATTTGGTCTCTGTGTGTAAACTAACCACG
GTAACCTGTGGCTAAAAGCGGAGAATAAGGCGCAACCCTATCAGCTTGTTGGTAGTGTAAAGGACT
ACCAAGGCCATGACGGGTAACGGGAAATCAGGGTTTGATTCCGGAGAGGGAGCCTGAGAGATGGC
TCCCACGTCCAAGGACGGCAGCAGGCGCGAAACTTGTCCACTCCTTACGGGGGAGACAGTCATGA
GACGTGAGTATAAGACCTGAGTGTAAAGACCTTAGGGTGAAGCAACTGGAGGGCAAGTCTGGT
>ZOTU716
GCAGGTTGATTCTGCCTGACGTAGATGCTAGTCTCTGAGATTAAGCCATGCATGTCAGTGAAGCCTT
ACGGCGGAACGGCGAACGGCTCAGTAATGTTGCGGTAATTTGGTCTCTGTGTGTAAACTAACCACG
GTAACCTGTGGCTAAAAGCGGAGAATAAGGCGCAACCCTATCAGCTTGTTGGTAGTGTAAAGGACT
ACCAAGGCCATGACGGGTAACGGGAAATCAGGGTTTGATTCCGGAGAGGGAGCCTGAGAGATGGC
TCCCACGTCCAAGGACGGCAGCAGGCGCGAAACTTGTCCACTCCTTACGGGGGAGACAGTCATGA
GACGTGAGTATAAGACCTGAGTGTAAAGACCTTAGGGTGAAGCAACTGGAGGGCAAGACTGGT
>ZOTU945
GCAGGTTGATTCTGCCTGACGTAGATGCTAGTCTCTGAGATTAAGCCATGCATGTCAGTGAAGCCTT
ACGGCGGAACGGCGAACGGCTCAGTAATGTTGCGGTAATTTGGTCTCTGTGTGTAAACTAACCACG
GTAACCTGTGGCTAAAAGCGGAGAATAAGGCGCAACCCTATCAGCTTGTTGGTAGTGTAAAGGACT
ACCAAGGCCATGACGGGTAACGGGAAATCAGGGTTTGATTCCGGAGAGGGAGCCTGAGAGATGGC
TCCCACGTCCAAGGACGGCAGCAGGCGCGAAACTTGTCCACTCCTTACGGGGGAGACAGTCATGA
GACGTGAGTATAAGACCTGAGTGTAAAGACCTTAGGGTGAAGCAATTGGAGGGCAAGTCTGGT
>ZOTU995
GCAGGTTGATTCTGCCTGACGTAGATGCTAGTCTCTGAGATTAAGCCATGCATGTCAGTGAAGCCTT
ACGGCGGAACGGCGAACGGCTCAGTAATGTTGCGGTAATTTGGTCTCTGTGTGTAAACTAACCACG
GTAACCTGTGGCTAAAAGCGGAGAATAAGGCGCAACCCTATCAGCTTGTTGGTAGTGTAAAGGACT
ACCAAGGCCATGACGGGTAACGGGAAATCAGGGTTTGATTCCGGAGAGGGAGCCTGAGAGATGGC
TCCCACGTCCAAGGACGGCAGCAGGCGCGAAACTTGTCCACTCCTTACGGGGGAGACAGTCATGA
GACGTGAGTATAAGACCTGAGTGTAAAGACCTTAGGGTGAAGCAATTGGAGGGCAAGACTGGT
>ZOTU999
TCAGGTTGATTCTGCCTGACGTAGATGCTAGTCTCTGAGATTAAGCCATGCATGTCAGTGAAGCCTT
ACGGCGGAACGGCGAACGGCTCAGTAATGTTGCGGTAATTTGGTCTCTGTGTGTAAACTAACCACG
GTAACCTGTGGCTAAAAGCGGAGAATAAGGCGCAACCCTATCAGCTTGTTGGTAGTGTAAAGGACT
ACCAAGGCCATGACGGGTAACGGGAAATCAGGGTTTGATTCCGGAGAGGGAGCCTGAGAGATGGC
TCCCACGTCCAAGGACGGCAGCAGGCGCGAAACTTGTCCACTCCTTACGGGGGAGACAGTCATGA
GACGTGAGTATAAGACCTGAGTGTAAAGACCTTAGGGTGAAGCAACTGGAGGGCAAGACTGGT
>ZOTU1039
TCAGGTTGATTCTGCCTGACGTAGATGCTAGTCTCTGAGATTAAGCCATGCATGTCAGTGAAGCCTT
ACGGCGGAACGGCGAACGGCTCAGTAATGTTGCGGTAATTTGGTCTCTGTGTGTAAACTAACCACG
GTAACCTGTGGCTAAAAGCGGAGAATAAGGCGCAACCCTATCAGCTTGTTGGTAGTGTAAAGGACT
ACCAAGGCCATGACGGGTAACGGGAAATCAGGGTTTGATTCCGGAGAGGGAGCCTGAGAGATGGC
TCCCACGTCCAAGGACGGCAGCAGGCGCGAAACTTGTCCACTCCTTACGGGGGAGACAGTCATGA
GACGTGAGTATAAGACCTGAGTGTAAAGACCTTAGGGTGAAGCAACTGGAGGGCAAGTCTGGT
>ZOTU1268
GCAGGTTGATTCTGCCTGACGTAGATGCTAGTCTCTGAGATTAAGCCATGCATGTCAGTGAAGCCTT
ACGGTGGAACGGCGAACGGCTCAGTAATGTTGCGGTAATTTGGTCTCTGTGTGTAAACTAACCACG
GTAACCTGTGGCTAAAAGCGGAGAATAAGGCGCAACCCTATCAGCTTGTTGGTAGTGTAAAGGACT
ACCAAGGCCATGACGGGTAACGGGAAATCAGGGTTTGATTCCGGAGAGGGAGCCTGAGAGATGGC

TCCCACGTCCAAGGACGGCAGCAGGCGCGAAACTTGTCCACTCCTTACGGGGGAGACAGTCATGA
GACGTGAGTATAAGACCTGAGTGTAAAGACCTTAGGGTGAAGCAACTGGAGGGCAAGACTGGT
>ZOTU1280
TCAGGTTGATTCTGCCTGACGTAGATGCTAGTCTCTGAGATTAAGCCATGCATGTCAGTGAAGCCTT
ACGGCGGAACGGCGAACGGCTCAGTAATGTTGCGGTAATTTGGTCTCTGTGTGTAAACTAACCACG
GTAACCTGTGGCTAAAAGCGGAGAATAAGGCGCAACCCTATCAGCTTGTTGGTAGTGTAAAGGACT
ACCAAGGCCATGACGGGTAACGGGAAATCAGGGTTTGATTCCGGAGAGGGAGCCTGAGAGATGGC
TCCCACGTCCAAGGACGGCAGCAGGCGCGAAACTTGTCCACTCCTTACGGGGGAGACAGTCATGA
GACGTGAGTATAAGACCTGAGTGTAAAGACCTTAGGGTGAAGCAATTGGAGGGCAAGTCTGGT
>ZOTU1289
GCAGGTTGATTCTGCCTGACGTAGATGCTAGTCTCTGAGATTAAGCCATGCATGTCAGTGAAGCCTT
ACGGTGGAACGGCGAACGGCTCAGTAATGTTGCGGTAATTTGGTCTCTGTGTGTAAACTAACCACG
GTAACCTGTGGCTAAAAGCGGAGAATAAGGCGCAACCCTATCAGCTTGTTGGTAGTGTAAAGGACT
ACCAAGGCCATGACGGGTAACGGGAAATCAGGGTTTGATTCCGGAGAGGGAGCCTGAGAGATGGC
TCCCACGTCCAAGGACGGCAGCAGGCGCGAAACTTGTCCACTCCTTACGGGGGAGACAGTCATGA
GACGTGAGTATAAGACCTGAGTGTAAAGACCTTAGGGTGAAGCAACTGGAGGGCAAGTCTGGT
>ZOTU1323
CCAGGTTGATTCTGCCTGACGTAGATGCTAGTCTCTGAGATTAAGCCATGCATGTCAGTGAAGCCTT
ACGGCGGAACGGCGAACGGCTCAGTAATGTTGCGGTAATTTGGTCTCTGTGTGTAAACTAACCACG
GTAACCTGTGGCTAAAAGCGGAGAATAAGGCGCAACCCTATCAGCTTGTTGGTAGTGTAAAGGACT
ACCAAGGCCATGACGGGTAACGGGAAATCAGGGTTTGATTCCGGAGAGGGAGCCTGAGAGATGGC
TCCCACGTCCAAGGACGGCAGCAGGCGCGAAACTTGTCCACTCCTTACGGGGGAGACAGTCATGA
GACGTGAGTATAAGACCTGAGTGTAAAGACCTTAGGGTGAAGCAACTGGAGGGCAAGTCTGGT
>ZOTU1436
TCAGGTTGATTCTGCCTGACGTAGATGCTAGTCTCTGAGATTAAGCCATGCATGTCAGTGAAGCCTT
ACGGCGGAACGGCGAACGGCTCAGTAATGTTGCGGTAATTTGGTCTCTGTGTGTAAACTAACCACG
GTAACCTGTGGCTAAAAGCGGAGAATAAGGCGCAACCCTATCAGCTTGTTGGTAGTGTAAAGGACT
ACCAAGGCCATGACGGGTAACGGGAAATCAGGGTTTGATTCCGGAGAGGGAGCCTGAGAGATGGC
TCCCACGTCCAAGGACGGCAGCAGGCGCGAAACTTGTCCACTCCTTACGGGGGAGACAGTCATGA
GACGTGAGTATAAGACCTGAGTGTAAAGACCTTAGGGTGAAGCAATTGGAGGGCAAGACTGGT
>ZOTU1467
CCAGGTTGATTCTGCCTGACGTAGATGCTAGTCTCTGAGATTAAGCCATGCATGTCAGTGAAGCCTT
ACGGCGGAACGGCGAACGGCTCAGTAATGTTGCGGTAATTTGGTCTCTGTGTGTAAACTAACCACG
GTAACCTGTGGCTAAAAGCGGAGAATAAGGCGCAACCCTATCAGCTTGTTGGTAGTGTAAAGGACT
ACCAAGGCCATGACGGGTAACGGGAAATCAGGGTTTGATTCCGGAGAGGGAGCCTGAGAGATGGC
TCCCACGTCCAAGGACGGCAGCAGGCGCGAAACTTGTCCACTCCTTACGGGGGAGACAGTCATGA
GACGTGAGTATAAGACCTGAGTGTAAAGACCTTAGGGTGAAGCAACTGGAGGGCAAGACTGGT
>ZOTU1545
TCAGGTTGATTCTGCCTGACGTAGATGCTAGTCTCTGAGATTAAGCCATGCATGTCAGTGAAGCCTT
ACGGTGGAACGGCGAACGGCTCAGTAATGTTGCGGTAATTTGGTCTCTGTGTGTAAACTAACCACG
GTAACCTGTGGCTAAAAGCGGAGAATAAGGCGCAACCCTATCAGCTTGTTGGTAGTGTAAAGGACT
ACCAAGGCCATGACGGGTAACGGGAAATCAGGGTTTGATTCCGGAGAGGGAGCCTGAGAGATGGC
TCCCACGTCCAAGGACGGCAGCAGGCGCGAAACTTGTCCACTCCTTACGGGGGAGACAGTCATGA
GACGTGAGTATAAGACCTGAGTGTAAAGACCTTAGGGTGAAGCAACTGGAGGGCAAGTCTGGT
>ZOTU1588
GCAGGTTGATTCTGCCTGACGTAGATGCTAGTCTCTGAGATTAAGCCATGCATGTCAGTGAAGCCTT
ACGGTGGAACGGCGAACGGCTCAGTAATGTTGCGGTAATTTGGTCTCTGTGTGTAAACTAACCACG
GTAACCTGTGGCTAAAAGCGGAGAATAAGGCGCAACCCTATCAGCTTGTTGGTAGTGTAAAGGACT
ACCAAGGCCATGACGGGTAACGGGAAATCAGGGTTTGATTCCGGAGAGGGAGCCTGAGAGATGGC

TCCCACGTCCAAGGACGGCAGCAGGCGCGAAACTTGTCCACTCCTTACGGGGGAGACAGTCATGA
GACGTGAGTATAAGACCTGAGTGTAAAGACCTTAGGGTGAAGCAATTGGAGGGCAAGTCTGGT
>ZOTU1732
CCAGGTTGATTCTGCCTGACGTAGATGCTAGTCTCTGAGATTAAGCCATGCATGTCAGTGAAGCCTT
ACGGCGGAACGGCGAACGGCTCAGTAATGTTGCGGTAATTTGGTCTCTGTGTGTAAACTAACCACG
GTAACCTGTGGCTAAAAGCGGAGAATAAGGCGCAACCCTATCAGCTTGTTGGTAGTGTAAAGGACT
ACCAAGGCCATGACGGGTAACGGGAAATCAGGGTTTGATTCCGGAGAGGGAGCCTGAGAGATGGC
TCCCACGTCCAAGGACGGCAGCAGGCGCGAAACTTGTCCACTCCTTACGGGGGAGACAGTCATGA
GACGTGAGTATAAGACCTGAGTGTAAAGACCTTAGGGTGAAGCAATTGGAGGGCAAGTCTGGT
>ZOTU1754
TCAGGTTGATTCTGCCTGACGTAGATGCTAGTCTCTGAGATTAAGCCATGCATGTCAGTGAAGCCTT
ACGGTGGAACGGCGAACGGCTCAGTAATGTTGCGGTAATTTGGTCTCTGTGTGTAAACTAACCACG
GTAACCTGTGGCTAAAAGCGGAGAATAAGGCGCAACCCTATCAGCTTGTTGGTAGTGTAAAGGACT
ACCAAGGCCATGACGGGTAACGGGAAATCAGGGTTTGATTCCGGAGAGGGAGCCTGAGAGATGGC
TCCCACGTCCAAGGACGGCAGCAGGCGCGAAACTTGTCCACTCCTTACGGGGGAGACAGTCATGA
GACGTGAGTATAAGACCTGAGTGTAAAGACCTTAGGGTGAAGCAATTGGAGGGCAAGACTGGT
>ZOTU1775
GCAGGTTGATTCTGCCTGACGTAGATGCTAGTCTCTGAGATTAAGCCATGCATGTCAGTGAAGCCTT
ACGGTGGAACGGCGAACGGCTCAGTAATGTTGCGGTAATTTGGTCTCTGTGTGTAAACTAACCACG
GTAACCTGTGGCTAAAAGCGGAGAATAAGGCGCAACCCTATCAGCTTGTTGGTAGTGTAAAGGACT
ACCAAGGCCATGACGGGTAACGGGAAATCAGGGTTTGATTCCGGAGAGGGAGCCTGAGAGATGGC
TCCCACGTCCAAGGACGGCAGCAGGCGCGAAACTTGTCCACTCCTTACGGGGGAGACAGTCATGA
GACGTGAGTATAAGACCTGAGTGTAAAGACCTTAGGGTGAAGCAATTGGAGGGCAAGACTGGT
>ZOTU1842
TCAGGTTGATTCTGCCTGACGTAGATGCTAGTCTCTGAGATTAAGCCATGCATGTCAGTGAAGCCTT
ACGGTGGAACGGCGAACGGCTCAGTAATGTTGCGGTAATTTGGTCTCTGTGTGTAAACTAACCACG
GTAACCTGTGGCTAAAAGCGGAGAATAAGGCGCAACCCTATCAGCTTGTTGGTAGTGTAAAGGACT
ACCAAGGCCATGACGGGTAACGGGAAATCAGGGTTTGATTCCGGAGAGGGAGCCTGAGAGATGGC
TCCCACGTCCAAGGACGGCAGCAGGCGCGAAACTTGTCCACTCCTTACGGGGGAGACAGTCATGA
GACGTGAGTATAAGACCTGAGTGTAAAGACCTTAGGGTGAAGCAATTGGAGGGCAAGTCTGGT
>ZOTU1874
CCAGGTTGATTCTGCCTGACGTAGATGCTAGTCTCTGAGATTAAGCCATGCATGTCAGTGAAGCCTT
ACGGCGGAACGGCGAACGGCTCAGTAATGTTGCGGTAATTTGGTCTCTGTGTGTAAACTAACCACG
GTAACCTGTGGCTAAAAGCGGAGAATAAGGCGCAACCCTATCAGCTTGTTGGTAGTGTAAAGGACT
ACCAAGGCCATGACGGGTAACGGGAAATCAGGGTTTGATTCCGGAGAGGGAGCCTGAGAGATGGC
TCCCACGTCCAAGGACGGCAGCAGGCGCGAAACTTGTCCACTCCTTACGGGGGAGACAGTCATGA
GACGTGAGTATAAGACCTGAGTGTAAAGACCTTAGGGTGAAGCAATTGGAGGGCAAGACTGGT
>ZOTU1912
TCAGGTTGATTCTGCCTGACGTAGATGCTAGTCTCTGAGATTAAGCCATGCATGTCAGTGAAGCCTT
ACGGTGGAACGGCGAACGGCTCAGTAATGTTGCGGTAATTTGGTCTCTGTGTGTAAACTAACCACG
GTAACCTGTGGCTAAAAGCGGAGAATAAGGCGCAACCCTATCAGCTTGTTGGTAGTGTAAAGGACT
ACCAAGGCCATGACGGGTAACGGGAAATCAGGGTTTGATTCCGGAGAGGGAGCCTGAGAGATGGC
TCCCACGTCCAAGGACGGCAGCAGGCGCGAAACTTGTCCACTCCTTACGGGGGAGACAGTCATGA
GACGTGAGTATAAGACCTGAGTGTAAAGACCTTAGGGTGAAGCAACTGGAGGGCAAGACTGGT
>ZOTU2452
CCAGGTTGATTCTGCCTGACGTAGATGCTAGTCTCTGAGATTAAGCCATGCATGTCAGTGAAGCCTT
ACGGTGGAACGGCGAACGGCTCAGTAATGTTGCGGTAATTTGGTCTCTGTGTGTAAACTAACCACG
GTAACCTGTGGCTAAAAGCGGAGAATAAGGCGCAACCCTATCAGCTTGTTGGTAGTGTAAAGGACT
ACCAAGGCCATGACGGGTAACGGGAAATCAGGGTTTGATTCCGGAGAGGGAGCCTGAGAGATGGC

TCCCACGTCCAAGGACGGCAGCAGGCGCGAAACTTGTCCACTCCTTACGGGGGAGACAGTCATGA
GACGTGAGTATAAGACCTGAGTGTAAAGACCTTAGGGTGAAGCAATTGGAGGGCAAGTCTGGT
>ZOTU2798
CCAGGTTGATTCTGCCTGACGTAGATGCTAGTCTCTGAGATTAAGCCATGCATGTCAGTGAAGCCTT
ACGGTGGAACGGCGAACGGCTCAGTAATGTTGCGGTAATTTGGTCTCTGTGTGTAAACTAACCACG
GTAACCTGTGGCTAAAAGCGGAGAATAAGGCGCAACCCTATCAGCTTGTTGGTAGTGTAAAGGACT
ACCAAGGCCATGACGGGTAACGGGAAATCAGGGTTTGATTCCGGAGAGGGAGCCTGAGAGATGGC
TCCCACGTCCAAGGACGGCAGCAGGCGCGAAACTTGTCCACTCCTTACGGGGGAGACAGTCATGA
GACGTGAGTATAAGACCTGAGTGTAAAGACCTTAGGGTGAAGCAACTGGAGGGCAAGTCTGGT
>ZOTU2932
CCAGGTTGATTCTGCCTGACGTAGATGCTAGTCTCTGAGATTAAGCCATGCATGTCAGTGAAGCCTT
ACGGTGGAACGGCGAACGGCTCAGTAATGTTGCGGTAATTTGGTCTCTGTGTGTAAACTAACCACG
GTAACCTGTGGCTAAAAGCGGAGAATAAGGCGCAACCCTATCAGCTTGTTGGTAGTGTAAAGGACT
ACCAAGGCCATGACGGGTAACGGGAAATCAGGGTTTGATTCCGGAGAGGGAGCCTGAGAGATGGC
TCCCACGTCCAAGGACGGCAGCAGGCGCGAAACTTGTCCACTCCTTACGGGGGAGACAGTCATGA
GACGTGAGTATAAGACCTGAGTGTAAAGACCTTAGGGTGAAGCAACTGGAGGGCAAGACTGGT

Capillaria hepatica (Nematoda)
>ZOTU59
AGTGGAGCATGCGGCTTAATTTGACTCAACACGGGAAAGCTCACCCGTCCCGAACACTGTCAGGAT
TGACAGATTGAGAGCTCTTTCTTGATTCAGTGGGTAGTGGTGCATGGCCGTTCTTAGTTGGTGGAGC
GATTTGTCTGGCCAATCCCGATAACGAACGAGACTCTGGCCTACTAAATAGTGACGGCTCAATTTGC
TTTGCCGAGCACTTCTTAGAGGGACAAGCGGCGGTACAAAGCCGCACGAGAAAGAGCAATAACAG
GTCTGTGATGCCCTTAGATGGACGGGGCTGCACGCGTGCTACACTGACGGCACCAGCGTGCGTTCA
AGCCTGTCTTGAAGAGGTCAGGTAATCGAATGAAATGTCCGTCGTGACTGGGACAGGGAATTGCA
>ZOTU122
AGTGGAGCATGCGGCTTAATTTGACTCAACACGGGAAAGCTCACCCGTCCCGAACACTGTCAGGAT
TGACAGATTGAGAGCTCTTTCTTGATTCAGTGGGTAGTGGTGCATGGCCGTTCTTAGTTGGTGGAGC
GATTTGTCTGGCCAATCCCGATAACGAACGAGACTCTGGCCTACTAAATAGTGACGGCTCAATTTGC
TTTGCCGAGCACTTCTTAGAGGGACAAGCGGCGGTACAAAGCCGCACGAGAAAGAGCAATAACAG
GTCTGTGATGCCCTTAGATGGACGGGGCTGCACGCGTGCTACACTGACGGCACCAGCGTGCGTTCA
AGCCTGTCTTGAAGAGGTCAGGTAATCGAATGAAATGTCCGTCGTGACTGGGATAGGGAATTGCA

Trichuris vulpis (Nematoda)
>ZOTU1396
AGTGGAGCATGCGGCTTAATTTGACTCAACACGGGAAAACTCACCCGTCCCGAACACTGTGAGGAT
TGACAGATCAAGAGCTCTTTCTTGATTCAGTGGGTAGTGGTGCATGGCCGTTCTTAGTTGGTGGAGC
GATTTGTCTGGCTAATTCCGATAACGAACGAGACTCTGGCCTACTAACTAGCGGCGGTGTTCATGCC
TCCTGACGGGGGCCGCGTGCGGCAACCGCCGGGCGCGCCCCTTGGAGCAGCAGCGCCGGCAGCCG
CTTCTTAGAGGGACCAGCGACACTTTCGCAAGCCGCACGAGAAAGAGCAATAACAGGTCTGTGAT
GCCCTTAGATGTACGGGGCTGCACGCGTGCTACACTGACGGCGTCAGCGTGCGTTCAAGCCCGGCC
TGGCAAGGTCGGGAAATCGGTTGAAACGTTCTCGTGACTGGGACAGGGAATTGCA
>ZOTU2965
AGTGGAGCATGCGGCTTAATTTGACTCAACACGGGAAAACTCACCCGTCCCGAACACTGTGAGGAT
TGACAGATCAAGAGCTCTTTCTTGATTCAGTGGGTAGTGGTGCATGGCCGTTCTTAGTTGGTGGAGC
GATTTGTCTGGCTAATTCCGATAACGAACGAGACTCTGGCCTACTAACTAGCGGCGGTGTTCATGCC
TCCTGACGGGGGCCGCGTGCGGCAACCGCCGGGCGCGCCCCTTGGAGCAGCAGCGCCGGCAGCCG
CTTCTTAGAGGGACCAGCGACACTTTCGCAAGCCGCACGAGAAAGAGCAATAACAGGTCTGTGAT
GCCCTTAGATGTACGGGGCTGCACGCGTGCTACACTGACGGCGTCAGCGTGCGTTCAAGCCCGGCC
TGGCAAGGTCGGGAAATCGGTTGAAACGTTCTCGTGACTGGGATAGGGAATTGCA

Pentatrichomonas hominis (Parabasalia)
>ZOTU30
TAGGCTATCACGGGTAACGGGCGGTTACCGTCGGACTGCCGGAGAAGGCGCCTGAGAGATAGCGA
CTATATCCACGGGTAGCAGCAGGCGCGAAACTTTCCCACTCGAGACTTTCGGAGGAGGTAATGACC
AGTTTCATGTGAAGCTTATGCTTCTGTGAATAGGATCACACTTTTCCAGTGTGGTGAAACCTAGCAG
AGGGCCAGTCTGGTGCCAGCAGCTGCGGTAATTCCAGCTCTGCGAGTTTGCTCCCATATTGTTGCAG
TTAAAACGCCCGTAGTCGGAATTGGACAGCAATGTCCCTACGTTTCAACGTTCACTGTGAACAAATC
AGGACGC

Isthmiophora hortensis (Platyhelminthes)
>ZOTU526
CAATTGGAGGGCAAGTCTGGTGCCAGCAGCCGCGGTAACTCCAGCTCCAGAAGCGTATATTAAAGT
TGTTGCAGTTAAAAAGCTCGTAGTTGGATCTGGGTTGCATGGCTACATGCCGTCGCTCGTGTGTCTG
CTACTACCGTGGCGGGCATATGAGTCGGTTTCGTGGTTGTGCTTCCTTTCTGCTGTGTCTGTTTACAG
GTGTCAGCGTGGTTGGTTGGCTTGCCTGCCGACCTGTTGGCATGCTTCTTGATGCCCTTAACCGGGT
GTCGGAGGCGGACAGCACGTTTACTTTGAACAAATCTGAGTGCTCAAAGCAGGCCTTTGTGCCTGA
AAGTTCTTGCATGGAATAATGGAATAGGACTTCGGTTCTATTTTGTTGGTTTTCGGATCCGAAGTAAT
GGTTAAGAGGGACAGACGGGGGCATTTGTATGGCGGTGTTAGAGGTGAAATTCTTGGATCGCCGCC
AGACAAACTACAGCGAAAGCA
>ZOTU726
CAATTGGAGGGCAAGTCTGGTGCCAGCAGCCGCGGTAACTCCAGCTCCAGAAGCGTATATTAAAGT
TGTTGCAGTTAAAAAGCTCGTAGTTGGATCTGGGTTGCATGGCTACATGCCGTCGCTCGTGTGTCTG
CTACTACCGTGGCGGGCATATGAGTCGGTTTCGTGGTTGTGCTTCCTTTCTGCTGTGTCTGTTTACAG
GTGTCAGCGTGGTTGGTTGGCTTGCCTGCCGACCTGTTGGCATGCTTCTTGATGCCCTTAACCGGGT
GTCGGAGGCGGACAGCACGTTTACTTTGAACAAATCTGAGTGCTCAAAGCAGGCCTTTGTGCCTGA
AAGTTCTTGCATGGAATAATGGAATAGGACTTCGGTTCTATTTTGTTGGTTTTCGGATCCGAAGTAAT
GGTTAAGAGGGACAGACGGGGGCATTTGTATGGCGGTGTTAGAGGTGAAATTCTTGGATCGCCGCC
AGACAAACTACTGCGAAAGCA
>ZOTU752
CAATTGGAGGGCAAGTCTGGTGCCAGCAGCCGCGGTAACTCCAGCTCCAGAAGCGTATATTAAAGT
TGTTGCAGTTAAAAAGCTCGTAGTTGGATCTGGGTTGCATGGCTACATGCCGTCGCTCGTGTGTCTG
CTACTACCGTGGCGGGCATATGAGTCGGTTTCGTGGTTGTGCTTCCTTTCTGCTGTGTCTGTTTACAG
GTGTCAGCGTGGTTGGTTGGCTTGCCTGCCGACCTGTTGGCATGCTTCTTGATGCCCTTAACCGGGT
GTCGGAGGCGGACAGCACGTTTACTTTGAACAAATCTGAGTGCTCAAAGCAGGCCTTTGTGCCTGA
AAGTTCTTGCATGGAATAATGGAATAGGACTTCGGTTCTATTTTGTTGGTTTTCGGATCCGAAGTAAT
GGTTAAGAGGGACAGACGGGGGCATTTGTATGGCGGTGTTAGAGGTGAAATTCTTGGATCGCCGCC
AGACAAACTAAAGCGAAAGCA
>ZOTU894
CAATTGGAGGGCAAGTCTGGTGCCAGCAGCCGCGGTAACTCCAGCTCCAGAAGCGTATATTAAAGT
TGTTGCAGTTAAAAAGCTCGTAGTTGGATCTGGGTTGCATGGCTACATGCCGTCGCTCGTGTGTCTG
CTACTACCGTGGCGGGCATATGAGTCGGTTTCGTGGTTGTGCTTCCTTTCTGCTGTGTCTGTTTACAG
GTGTCAGCGTGGTTGGTTGGCTTGCCTGCCGACCTGTTGGCATGCTTCTTGATGCCCTTAACCGGGT
GTCGGAGGCGGACAGCACGTTTACTTTGAACAAATCTGAGTGCTCAAAGCAGGCCTTTGTGCCTGA
AAGTTCTTGCATGGAATAATGGAATAGGACTTCGGTTCTATTTTGTTGGTTTTCGGATCCGAAGTAAT
GGTTAAGAGGGACAGACGGGGGCATTTGTATGGCGGTGTTAGAGGTGAAATTCTTGGATCGCCGCC
AGACAAACTAATGCGAAAGCA

Supplementary References

Alvarez-Pellitero, M.-P., Gonzalez-Lanza, M.-C., 1986. *Eimeria* spp. from cyprinid fish of the Duero basin (north-west Spain). J. Fish Dis. 9, 325–336.

Amer, S., Kim, S., Han, J.-I., Na, K.-J., 2019. Prevalence and genotypes of *Enterocytozoon bieneusi* in wildlife in Korea: A public health concern. Parasites Vectors 12, 160.

Augustine, P.C., Danforth, H.D., 1990. Avian *Eimeria*: Invasion in foreign host birds and generation of partial immunity against coccidiosis. Avian Dis. 34, 196–202.

Chai, J.-Y., Jung, B.-K., 2019. Epidemiology of trematode infections: An update, in: Toledo, R., Fried, B. (Eds.), Digenetic Trematodes. Springer, Switzerland, pp. 359–409.

Choe, S., Na, K.-J., Kim, Y., Jeong, D.-H., Yang, J.-J., Eom, K.S., 2019. Infections of two *Isthmiophora* species (Digenea: Echinostomatidae) in wild mammals from Republic of Korea with their morphological descriptions. Korean J. Parasitol. 57, 647–656.

Diakou, A., Migli, D., Dimzas, D., Morelli, S., Di Cesare, A., Youlatos, D., Lymberakis, P., Traversa, D., 2021. Endoparasites of European wildcats (*Felis silvestris*) in Greece. Pathogens 10, 594.

Dunn, J.J., Columbus, S.T., Aldeen, W.E., Davis, M., Carroll, K.C., 2002. *Trichuris vulpis* recovered from a patient with chronic diarrhea and five dogs. J. Clin. Microbiol. 40, 2703–2704.

Ebmer, D., Wiedermann, S., Sattmann, H., 2020. Morphological identifications of gastrointestinal nematodes and acanthocephalans of raptors and owls from Austria. Ann. Naturhist. Mus. Wien, B 122, 169–174.

Edgar, R.C., 2004. MUSCLE: a multiple sequence alignment method with reduced time and space complexity. BMC Bioinformatics 5, 113.

Fuehrer, H.-P., 2014a. An overview of the host spectrum and distribution of *Calodium hepaticum* (syn. *Capillaria hepatica*): Part 1—Muroidea. Parasitol. Res. 113, 619–640.

Fuehrer, H.-P., 2014b. An overview of the host spectrum and distribution of *Calodium hepaticum* (syn. *Capillaria hepatica*): Part 2—Mammalia (excluding Muroidea). Parasitol. Res. 113, 641–651.

Guzeeva, E.A., Spiridonov, S.E., 2013. Molecular-taxonomic study of two species of *Pseudonymus* Diesing, 1857 (Oxyuridomorpha: Thelastomatoidea: Pseudonymidae) from water beetles (Coleóptera: Hydrophilidae) of Volga estuary. Russ. J. Nematol. 21, 131–139.

Honisch, M., Krone, O., 2008. Phylogenetic relationships of Spiruromorpha from birds of prey based on 18S rDNA. J. Helminthol. 82, 129–133.

Hughes, A.L., Piontkivska, H., 2003. Phylogeny of Trypanosomatidae and Bodonidae (Kinetoplastida) based on 18S rRNA: Evidence for paraphyly of *Trypanosoma* and six other genera. Mol. Biol. Evol. 20, 644–652.

Hughes, M.R., Gibson, A.A., Wolfe, E.R., Bronson, C.D., Duffield, D.A., 2021. Phylogenetics and genetic variation of *Heligmosomoides thomomyos* in Western pocket gophers (*Thomomys* spp.). J. Nematol. 53, e2021-2110.

Katherine Lynn, C., Robert Byron, B., 2019. *Heterakis gallinarum*, the cecal nematode of gallinaceous birds: A critical review. Avian Dis. 63, 381–388.

Li, X., Li, J., Zhang, X., Yang, Z., Yang, J., Gong, P., 2017. Prevalence of *Pentatrichomonas hominis* infections in six farmed wildlife species in Jilin, China. Vet. Parasitol. 244, 160–163.

Lipa, J.J., 1966. Miscellaneous observations on protozoan infections of *Nepa cinerea* Linnaeus including descriptions of two previously unknown species of *Microsporidia*, *Nosema bialoviesianae* sp. n. and *Thelohania nepae* sp.n. J. Invertebr. Pathol. 8, 158–166.

Lo, C.L.C., Fernandez, D.A.P., de Luna, M.C.T., de Guia, A.P.O., Paller, V.G.V., 2021. Diet, parasites, and other pathogens of Sunda leopard cats (*Prionailurus javanensis* Desmarest 1816) in Aborlan, Palawan Island, Philippines. J. Parasit. Dis. 45, 627–633.

Maritz, J.M., Land, K.M., Carlton, J.M., Hirt, R.P., 2014. What is the importance of zoonotic trichomonads for human health? Trends Parasitol. 30, 333–341.

Ogilvie, B.M., Jones, V.E., 1971. *Nippostrongylus brasiliensis*: A review of immunity and the host/parasite relationship in the rat. Exp. Parasitol. 29, 138–177.

Okada, H., Kadota, I., 2003. Host status of 10 fungal isolates for two nematode species, *Filenchus misellus* and *Aphelenchus avenae*. Soil Biol. Biochem. 35, 1601–1607.

Paradis, E., Claude, J., Strimmer, K., 2004. APE: Analyses of Phylogenetics and Evolution in R language. Bioinformatics 20, 289–290.

Rosenthal, B.M., Dunams-Morel, D., Ostoros, G., Molnár, K., 2016. Coccidian parasites of fish encompass profound phylogenetic diversity and gave rise to each of the major parasitic groups in terrestrial vertebrates. Infect. Genet. Evol. 40, 219–227.

Saitou, N., Nei, M., 1987. The neighbor-joining method: a new method for reconstructing phylogenetic trees. Mol. Biol. Evol. 4, 406–425.

Sanmartin, M.L., Alvarez, F., Barreiro, G., Leiro, J., 2004. Helminth fauna of Falconiform and Strigiform birds of prey in Galicia, Northwest Spain. Parasitol. Res. 92, 255–263.

Santin, M., Fayer, R., 2011. Microsporidiosis: *Enterocytozoon bieneusi* in domesticated and wild animals. Res. Vet. Sci. 90, 363–371.

Smith, P.E., 1953. Life history and host-parasite relations of *Heterakis spumosa*, a nematode parasite in the colon of the rat. Am. J. Hyg. 57, 194–221.

Stensvold, C.R., Lebbad, M., Victory, E.L., Verweij, J.J., Tannich, E., Alfellani, M., Legarraga, P., Clark, C.G., 2011. Increased sampling reveals novel lineages of *Entamoeba*: Consequences of genetic diversity and host specificity for taxonomy and molecular detection. Protist 162, 525–541.
